# Proton pump inhibitor usage is associated with higher all-cause mortality and CV events in peritoneal dialysis patients

**DOI:** 10.1080/0886022X.2022.2043903

**Published:** 2022-03-02

**Authors:** Yingsi Zeng, Lingling Liu, Liya Zhu, Xiaojiang Zhan, Fenfen Peng, Xiaoran Feng, Qian Zhou, Yujing Zhang, Zebin Wang, Jianbo Liang, Jiao Li, Yueqiang Wen

**Affiliations:** a Department of Nephrology, The Second Affiliated Hospital, Guangzhou Medical University, Guangzhou, China; b Department of General Medicine, The Third Affiliated Hospital, Sun Yat-sen University, Guangzhou, China; c Department of Nephrology, The First Affiliated Hospital of Nanchang University, Nanchang, Jiangxi, China; d Department of Nephrology, Zhujiang Hospital, Southern Medical University, Guangzhou, China; e Department of Nephrology, Jiujiang NO.1 People’s Hospital, Jiangxi, China; f Department of Medical Statistics, Clinical Trials Unit, The First Affiliated Hospital, Sun Yat-sen Univeristy, Guangzhou, China; g Department of Cardiology, The Second Affiliated Hospital, Guangzhou Medical University, Guangzhou, China

**Keywords:** Peritoneal dialysis, proton pump inhibitors, all-cause mortality, cardiovascular event

## Abstract

**Objectives:**

A long period of inappropriate proton pump inhibitors (PPI) treatment has been proved to be associated with adverse prognosis in general population and hemodialysis patients. This study was conducted to clarify the impact of PPI usage on mortality and adverse cardiovascular (CV) events in peritoneal dialysis (PD) patients.

**Methods and design:**

This is a retrospective study. A total of 905 patients were enrolled from two PD centers, including 211 patients on PPI treatment and 618 patients not on PPIs. Kaplan–Meier curves were used to identify the incidence of adverse outcomes. Multivariate Cox regression models and inverse probability of treatment weighting (IPTW) were applied to analyze hazard ratios (HRs) for adverse outcomes.

**Results:**

During follow-up, 162 deaths and 102 CV events were recorded. Kaplan–Meier curve demonstrated all-cause mortality (log-rank test *p* = .018) and CV events (log-rank test *p* = .024) were significantly higher in PPI usage group. Multivariate Cox regression models and IPTW showed that PPI usage was an indicator for all-cause mortality (HR = 1.35, 95%CI = 1.09–1.67, *p* = .006) and CV events (HR = 1.78, 95%CI = 1.35–2.32, *p* < .001).

**Conclusions:**

PPI usage is associated with higher all-cause mortality and CV events in PD patients. Clinicians are supposed to be more careful when using PPI and need to master the indications more rigorously in patients receiving PD treatment.

## 1. Introduction

Proton pump inhibitors (PPIs) are currently one of the most commonly prescribed medications. Recently, emerging evidences suggested that PPIs have been overprescribed. Investigation demonstrated that 25–70% of patients using PPIs do not have appropriate indications in the United States [1]. Considerable studies showed that PPI usage is associated with adverse events, such as dementia [2], fractures [3], hypomagnesemia [4], vitamin B12 deficiency [5], and cardiovascular (CV) events [6,7]. Besides, PPI usage also indicated adverse kidney outcomes, accelerating the progression of chronic kidney diseases [8].

As CV events are the main complications, anti-platelet drugs are widely used to treat CV diseases (CVD) in patients with end-stage kidney disease (ESKD) [9]. Accordingly, PPIs are also widely used to avoid gastrorrhagia induced by anti-platelet drugs. Over the past years, more and more reports have linked the long-term use of PPI to various CV and non-CV adverse reactions. Therefore, in this article necessary studies were conducted to check whether it is appropriate to take PPIs for a long period in ESKD patients. In recent studies, PPI usage was found to be closely associated with mortality in general population and hemodialysis (HD) patients [10]. de Francisco et al. [11] analyzed 2222 HD patients including 1776 on PPI therapy and 466 patients not on PPI, where it was found that PPI usage was associated with all-cause mortality. Up till now, the relationship between PPI usage and mortality as well as CV events have not been reported in peritoneal dialysis (PD) patients.

Therefore, this study was conducted to clarify the impact of PPI usage on mortality and CV events in PD patients.

## 2. Materials and methods

### 2.1. Subjects

**Figure 1.**
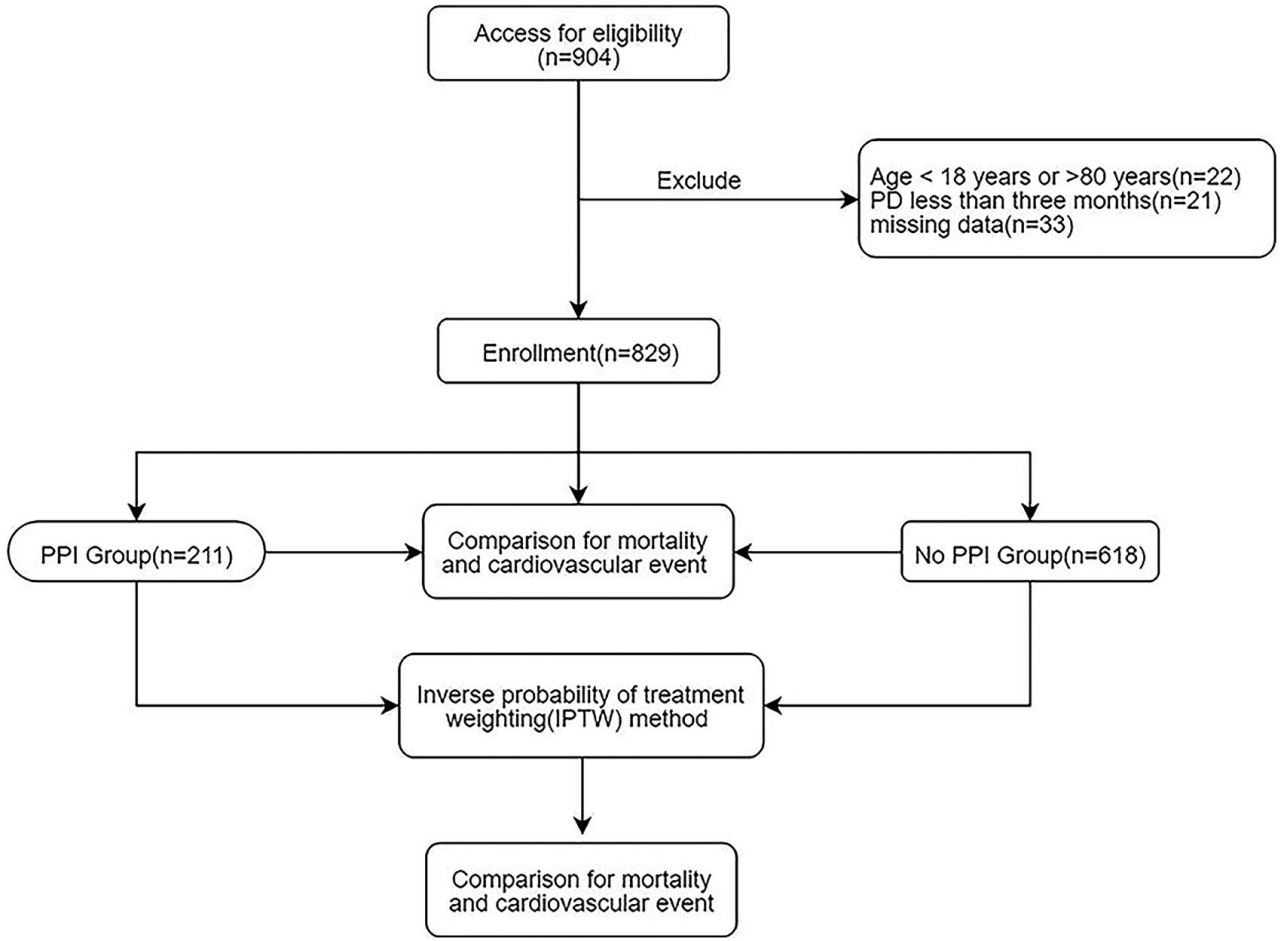
Flow chart including patient enrollment and outcomes.

This retrospective multicenter study was conducted to evaluate the effect of PPI usage on CV events and mortality in PD patients. This study included 905 PD patients from the Second Affiliated Hospital of Guangzhou Medical University and Zhujiang Hospital Affiliated to Southern Medical University from 1 January 2010 to 31 December 2016. The median follow-up interval was 43.759 ± 0.875 months. Patients were divided into the PPI group (use of PPIs at baseline) and non-PPI group. Patients using PPI for more than 1 week continuously were included in the PPI group [12]. Patients were excluded for the following reasons: age younger than 18 years or older than 80 years (*n* = 22), PD was maintained for less than 3 months (*n* = 21), missing data (*n* = 33). In all, 829 patients were enrolled in the study, including 211 on PPI therapy and 618 not receiving PPI (Figure 1). All procedures performed in studies involving human participants were in accordance with the ethical standards of the institutional, and with the 1964 Helsinki Declaration and its later amendments or comparable ethical standards. This study was warranted by the Institutional Review Board of two PD centers (the approval number was 2021-hg-ks-15). As the existing medical records were collected, written informed consent was not required.

### 2.2. Study outcome

All patients were followed up until CV events or death, transferring to HD therapy, transferring to kidney transplantation, transferring of care to other centers, lost to follow up or censoring on 31 December 2017. The primary outcome was all-cause mortality, while the secondary outcome was CV events. CVD was defined as recording of any of the following conditions in the patient’s medical records: coronary heart disease, coronary atherosclerotic heart disease, acute myocardial infarction, cardiac arrest, cerebrovascular accident, stroke, and congestive heart failure.

### 2.3. Clinical data

The baseline demographic data included center, age, gender, and comorbid (history of hypertension, diabetes, CVD, and gastrointestinal bleeding). Baseline data were collected within 3 months of the initiation of PD. Clinical biochemical indicators included body mass index (BMI), systolic blood pressure (SBP), diastolic blood pressure (DBP), medication history (including calcium channel blockers (CCB), angiotensin converting enzyme inhibitor (ACEI), angiotensin receptor blocker (ARB) (β-blockers, aspirin, statins), hemoglobin, creatinine, urea nitrogen, uric acid, fasting blood glucose (FBG), cholesterol, triglycerides, calcium, potassium, phosphorus, total Kt/v, residual renal function (RRF). Patients who reported current use of insulin or oral hypoglycemic agents and/or who had a clinical diagnosis of type 1 or type 2 diabetes mellitus were considered to have diabetes mellitus. Hypertension was recorded if the patient took antihypertensive drugs or had two separate blood pressure measurements ≥140/90 mmHg. CV events were defined as recording of any of the following conditions in the patient’s medical records: coronary heart disease, coronary atherosclerotic heart disease, acute myocardial infarction, congestive heart failure, cardiac arrest, cerebrovascular accident, stroke, cerebral infarction, cerebral hemorrhage. The types of PPI used included lansoprazole, omeprazole, esomeprazole, rabeprazole, and pantoprazole. It was not possible to record the total duration and the total dose used by patients as keeping track of their prescriptions after discharge from hospital was difficult.

Laboratory measurements were obtained using standard methods in the clinical laboratory. Total Kt/V was calculated using PD Adequest software 2.0 (Baxter, Deerfield, IL). Medicine usage was recorded based on prescriptions. Patients returned to these centers for quarterly evaluation, and trained nurses interviewed the patients on telephone monthly to assess general conditions.

### 2.4. Statistical analyses

Continuous variables were described as mean ± standard deviation (SD) or median (25th–75th percentile), and categorical data were given as number (percentages). Comparisons of the variable between groups were performed using the *t* test for normally distributed variables and Mann–Whitney *U* test for skewed continuous variables. Differences among the two groups were tested using Chi-square test for categorical variables. Mortality and incidence of CV events were calculated using the Kaplan–Meier curve and differences among distributions were assessed by log-rank test. Cox regression models were used to evaluate the relationship among PPI usage with mortality and CV events in patients undergoing PD, initially without adjustment and subsequently by adjusting several groups of covariates. Inverse probability of treatment weighting (IPTW) analysis was applied to assess the influence of PPI usage. IPTW analysis was obtained by using the propensity scores of all indicators before matching, and statistically adjusting the covariates to reduce the bias, thereby achieving randomization. Variables included in the IPTW analysis were center, age, sex, BMI, diabetes mellitus, hypertension, systolic pressure, history of CVD, history of gastrointestinal bleeding, usage of ACEI/ARB, β-blcoker, CCB, aspirin, and statins, hemoglobin, creatinine, urea nitrogen, cholesterol, triglycerides, uric acid, calcium, potassium, phosphorus, and Kt/V, RRF.

In Cox regression models, time at risk was from study entry until CV events, death, transferring to HD therapy, transferring to kidney transplantation, transferring of care to other centers, or the end of study on 31 December 2017. Missing data were filled by miss Forest method. Statistical analysis was completed by SPSS 23.0 and R software (version R-3.6.2, www.r-project.org). All tests were performed bilaterally, and *p* < .05 was considered to be statistically significant.

## 3. Results

During follow-up, 162 deaths and 102 CV events were recorded, the cause of death included CVD (*n* = 65), infection (*n* = 32), gastrointestinal bleeding (*n* = 5), malignant tumor (*n* = 8), uremic encephalopathy (*n* = 25), and others (*n* = 27). Baseline characteristics of the cohort are shown in Table 1. Median age was 53 (42, 63), of which 463 were male and 366 were female. A total of 212 (25.6%) patients had a history of diabetes, 179 (21.6%) patients had a history of CVD, while 58 (7.0%) patients had a history of gastrointestinal bleeding. Patients in PPI group were older, and more often suffered from diabetes and CVD than the non-PPI group.

**Table 1.** Patient demographic and clinical characteristics.

	Group 1 Non-PPI (*n* = 618)	Group 2 PPI (*n* = 211)	*p* Value
No. of C1/C2	308/310	46/165	<.001
No. of men/women	361/257	102/109	.011
Follow-up period	42.7 (25.6, 64.9)	33.4 (20.6, 47.3)	<.001
Demographics			
Age (y)	52.0 (42.0, 63.0)	56.0 (45.0, 65.0)	.012
BMI (kg/m^2^)	22.6 (20.6, 25.0)	22.5 (20.7, 24.5)	.573
Protopathy			
Chronic glomerulonephritis	302 (48.9%)	105 (49.8%)	.731
Diabetic nephropathy	125 (20.2%)	46 (21.8)	.526
Hypertensive nephropathy	102 (16.5%)	25 (11.8)	.147
Lupus nephritis	1 (0.2%)	4 (1.9%)	.005
Anaphylatic purpura nephritis	1 (0.2%)	2 (0.9%)	.101
ANCA-related nephritis	1 (0.2%)	1 (0.5%)	.425
Interstitial nephritis	25 (4.0%)	5 (2.4%)	.069
Polycystic kidney	8 (1.3%)	8 (3.8%)	.113
Obstructive nephropathy	52 (8.4%)	15 (7.1%)	.082
Plasma cell nephropathy	1 (0.2%)	0 (0%)	.552
Comorbid			
Diabetes	145 (23.5%)	67 (31.8%)	.017
Hypertension	310 (50.2%)	152 (72.0%)	<.001
SBP (mm Hg)	136 (144, 160)	151 (133, 171)	.098
DBP (mm Hg)	85 (79, 93)	84 (77, 96)	.236
Cardiovascular disease	120 (19.4%)	59 (28.0%)	.009
Gastrointestinal bleeding	35 (5.7%)	23 (10.9%)	.010
Laboratory variables			
Hemoglobin (g/L)	96.0 (84.0, 110.0)	90.0 (82.0, 104.0)	.009
Creatinine (μmol)	751.5 (549.0, 977.8)	799.0 (568.0, 1104.0)	.054
Urea nitrogen (mmol/L)	18.9 (14.6, 24.7)	18.9 (13.1, 24.0)	.256
Uric acid (mmol/L)	434.0 (371.0, 491.0)	412.0 (359.0, 479.0)	.034
FBG (mmol/L)	4.6 (4.1, 5.6)	4.5 (3.8, 5.9)	.145
Cholesterol (mmol/L)	4.4 (3.9, 5.0)	4.4 (3.8, 5.3)	.437
Triglycerides (mmol/L)	1.4 (1.0, 2.0)	1.5 (1.1, 2.3)	.069
Calcium (mmol/L)	2.1 (2.0, 2.3)	2.0 (1.9, 2.2)	<.001
Potassium (mmol/L)	3.9 (3.4, 4.4)	3.9 (3.4, 4.4)	.941
Phosphorus (mmol/L)	1.5 (1.2, 1.9)	1.6 (1.2, 2.0)	.353
Total Kt/V	2.3 (1.9, 2.7)	2.2 (1.8, 2.6)	.495
RRF (mL/min)	7.0 (2.6, 26.2)	17.4 (4.1, 34.3)	<.001
Treatments			
CCB	519 (84.0%)	180 (85.3%)	.647
β-blocker	322 (52.1%)	111 (52.6%)	.899
ACEI/ARB	348 (56.3%)	95 (45.0%)	.005
Aspirin	76 (12.3%)	19 (9.0%)	.195
Statins	93 (15.0%)	56 (26.5%)	<.001

Note: All continuous variables are skewed distribution – the values for continuous variables are given as median (P25,P75).

PPI: proton pump inhibitor; C1: center 1; C2: center 2; BMI: body mass index; FBG: fasting blood-glucose; Kt/V: K – dialyzer clearance of urea, t – dialysis time, V – volume of distribution of urea; RRF: residual renal function; CCB: calcium channel blocker; ACEI: angiotensin converting enzyme inhibitors; ARB: angiotensin receptor blocker.

The significant risk factors for patients with the higher incidence of new-onset CV event as well as all-cause mortality are given in Table 2 by adjusting for covariates (*p* < .05 univariable logistic regression). Higher mortality was associated with center, older age, female, history of hypertension, history of diabetes, history of CVD, and usage of β-blocker (Table 2). Higher incidence of CV event was associated with older age, lower blood glucose level, history of hypertension, usage of aspirin, usage of β-blocker, and SBP (Table 2).

**Table 2.** Significant risk factors for all-cause mortality and CV events.

Risk factors	Univariable logistic regression		Multivariable logistic regression	
	HR (95%CI)	*p* Value	HR (95%CI)	*p* Value
All-cause mortality				
C2 vs C1	0.43 (0.31–0.62)	<.001	0.86 (0.79–0.94)	.001
Sex ( female vs male)	2.13 (1.51–3.03)	<.001	2.20 (1.45–3.33)	<.001
Age (years)	1.09 (1.07–1.10)	<.001	1.06 (1.04–1.08)	<.001
Diabetes (yes vs no)	6.21 (4.29–8.98)	<.001	2.69 (1.70–4.25)	<.001
Hypertension (yes vs no)	5.20 (3.35–8.07)	<.001	2.03 (1.20–3.45)	.009
CVD history (yes vs no)	5.81 (3.99–8.45)	<.001	2.23 (1.41–3.54)	.001
β-blocker (yes vs no)	1.42 (1.01–2.02)	.047	1.78 (1.17–2.71)	.007
Aspirin (yes vs no)	5.85 (3.73–9.18)	<.001		
DBP (mm Hg)	0.96 (0.94–0.97)	<.001		
Creatinine (umol/L)	0.998 (0.998–0.999)	<.001		
Urea nitrogen (mmol/L)	0.95 (0.93–0.97)	<.001		
FBG (mmol/L)	1.23 (1.15–1.31)	<.001		
Cholesterol (mmol/L)	1.31 (1.13–1.51)	<.001		
Potassium (mmol/L)	0.60 (0.46–0.76)	<.001		
ACEI/ARB (yes vs no)	1.57 (1.11–2.24)	.012		
Calcium (mmol/L)	2.00 (1.04–3.97)	.047		
Uric acid (mmol/L)	0.998 (0.996–1.00)	.028		
Phosphorus (mmol/L)	0.72 (0.53–0.97)	.032		
RRF (mL/min)	0.99 (0.98–1.00)	.047		
CV events				
Age (years)	1.06 (1.04–1.08)	<.001	1.04 (1.02–1.06)	<.001
Hypertension (yes vs no)	5.44 (3.09–9.60)	<.001	3.49 (1.89–6.45)	<.001
Aspirin (yes vs no)	5.46 (3.35–8.89)	<.001	2.20 (1.23–3.92)	.008
SBP (mm Hg)	0.99 (0.98–1.00)	.029	0.99 (0.98–1.00)	.037
FBG (mmol/L)	1.21 (1.13–1.30)	<.001	1.13 (1.04–1.22)	.003
β-blocker (yes vs no)	1.63 (1.07–2.50)	.024	2.02 (1.26–3.24)	.004
DBP (mm Hg)	0.96 (0.94–0.98)	<.001		
C2 vs C1	0.50 (0.33–0.76)	.001		
ACEI/ARB (yes vs no)	1.97 (1.27–3.06)	.002		
CVD history (yes vs no)	3.33 (2.16–5.14)	<.001		
Diabetes (yes vs no)	3.52 (2.30–5.38)	<.001		

PPI: proton pump inhibitor; C1: center 1; C2: center 2; CVD: cardiovascular disease; BP: blood pressure; FBG: fasting blood glucose; ACEI: angiotensin converting enzyme inhibitors; ARB: angiotensin receptor blocker; RRF: residual renal function.

**Figure 2.**
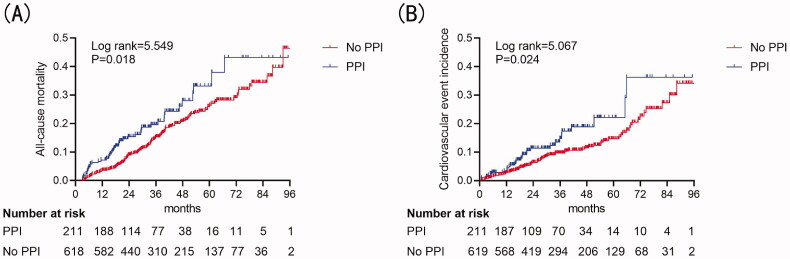
Cumulative incidence curves for mortality and CV events by category of the use of PPI. (A) Cumulative incidence curves for all-cause mortality. (B) Cumulative incidence curves for the incidence of CV events. The curves were constructed using the Kaplan–Meier method and compared using the Mantel–Cox log-rank test.

**Figure 3.**
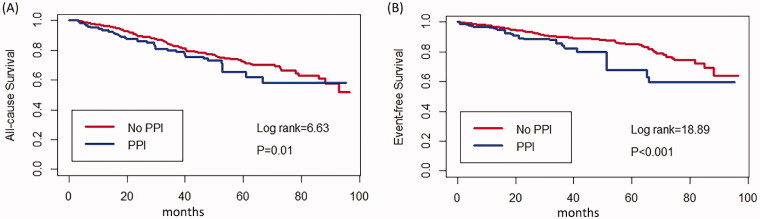
Cumulative incidence curves for mortality and CV events by category of the usage of PPI after IPTW. (A) Cumulative incidence curves for all-cause survival. (B) Cumulative incidence curves for the incidence of CV events. The curves were constructed using the Kaplan–Meier method and compared using the Mantel–Cox log-rank test.

Kaplan–Meier cumulative incidence curve demonstrated that all-cause mortality (log-rank test *p* = .018) (Figure 2(A)) and the incidence of CV events (log-rank test *p* = 0.024) (Figure 2(B)) were significantly higher in the PPI group. Besides, Kaplan–Meier cumulative incidence curve after IPTW also showed a significant association among all-cause mortality (log-rank test *p* = .01) (Figure 3(A)) and the incidence of CV events (log-rank test *p* < .001) (Figure 3(B)) in the PPI group. Multivariate Cox regression models showed that PPI was an independent risk factor for all-cause mortality (HR = 1.47, 95%CI = 1.02–2.13, *p* = .042) and CV events (HR = 1.80, 95%CI = 1.13–2.87, *p* = .014) in PD patients after adjusting for complications, medication, age, sex, center, and biochemical examination. IPTW method also confirmed PPI usage as a predictor for all-cause mortality (HR = 1.35, 95%CI = 1.09–1.67, *p* = .006) and CV events (HR = 1.78, 95%CI = 1.35–2.32, *p* < .001) (Table 3).

**Table 3.** Relationship between PPI and the adverse prognosis.

	HR (95%CI)	*p* Value
All-cause mortality		
Unadjusted	1.52 (1.07–2.15)	.019
Model 1	1.67 (1.15–2.41)	.006
Model 2	1.46 (1.01–2.10)	.044
Model 3	1.47 (1.02–2.13)	.042
IPTW	1.35 (1.09–1.67)	.006
New-onset CVE		
Unadjusted	1.64 (1.06–2.54)	.026
Model 1	1.89 (1.19–3.00)	.007
Model 2	1.72 (1.08–2.74)	.022
Model 3	1.80 (1.13–2.87)	.014
IPTW	1.78 (1.35–2.32)	<.001

Model 1: sex, age, center.

Model 2:

All-cause mortality: Model 1 plus comorbid conditions (diabetes, SBP, history of CVD, history of gastrointestinal bleeding, β-blocker).

New-onset CVE: Model 1 plus comorbid conditions (diabetes, SBP, history of gastrointestinal bleeding, β-blocker).

Model 3: Model 2 plus uric acid, total cholesterol, Kt/V, RRF.

IPTW: inverse probability of treatment weighted method; PPI: proton pump inhibitor; HR: hazard ratio; CI: confidence interval; CVD: cardiovascular disease; CVE: cardiovascular event; SBP: systolic pressure; Kt/V: K – dialyzer clearance of urea, t – dialysis time, V – volume of distribution of urea; RRF: residual renal function.

Note: Reference group is the non-PPI group.

## 4. Discussion

In this retrospective multicenter study of 905 PD patients, it was found that PPI usage was related to all-cause mortality and CV events. PPI is widely used around the world to treat and prevent gastrointestinal bleeding, but its safety and indication have never been clarified. Plenty of studies have proposed that PPI usage results in increased adverse prognosis in both general population and ESKD patients. Xie et al. [10] concluded that PPI was related to the increased risk of death in the general population. Chen et al. [13] showed the result of a retrospective study, with the aim of disclosing the effect of concomitant use, suggesting that PPI usage was associated with death. de Francisco et al. [11] analyzed 2222 HD patients including 1776 on PPI therapy and 466 patients not on PPI, where an association between PPI usage and all-cause mortality in HD patients was reported. More recently, a prospective multicenter observational study of 367 patients by Kosedo et al. [14] indicated that the usage of PPI in HD patients increased the risk of mortality and CV events. However, to the best of the authors’ knowledge, fewer studies have reported the relationship between adverse prognosis and PPI usage in PD patients.

This study suggested that PPI was an independent risk factor for all-cause mortality in PD patients. The result was consistent with other studies in general population and HD patients. Several reasons are considered to be related to the results. First of all, experiments in mice have shown that the application of PPI upregulates mRNA and protein expression and leads to increased heme oxygenase-1 enzyme activity in gastric and renal cells [15]. Heme oxygenase-1 is generally considered to be beneficial; however, when PPI is applied, it will destroy the acidification and protein stabilization of lysosomes and cause oxidative stress, dysfunction [16], as well as promotes the aging of endothelial cell [17]. Secondly, PPI usage has been proved to be related to hypomagnesemia in not only general population but also in HD patients [18–20]. There are increasing evidences which indicate that hypomagnesemia might accelerate mortality among HD patients [21,22]. On one hand, it was reported that magnesium deficiency was associated with insulin resistance and metabolic syndrome [23]. Magnesium is an important cofactor for many enzymes involved in glucose metabolism, while metabolic syndrome has been found to be a predictor of mortality hazard [24]. Magnesium is closely related to the immune system in both nonspecific and specific immune responses (also known as innate and acquired immune responses), and is involved in a variety of immune responses such as immune globulin synthesis, C3 converting enzymes, and immune cell adherence [25]. Unfortunately, serum magnesium concentration was not available in the database owing to which the above inferences could not be verified in this study. Thirdly, the usage of PPI had also been correlated to a higher incidence of bone fracture [3]. PPI is an effective gastric acid secretion blocker, which is believed to be necessary to absorb calcium. PPI can reduce the absorption of alkaline calcium and even lead to a decrease in bone mineral density, further resulting in bone fracture. A previous study by Kiadaliri et al. [26] reported that fracture was mentioned as a contributory cause of death. Potential comorbidities are likely to affect long-term risk of death. Bone fracture leads to a series of complications such as infection and embolism, which gradually occur due to the long-term bed rest affecting the quality of life, even leading to increased risk of death.

The results of this study also indicated that PPI usage was an independent risk factor for CV events in PD patients. PPIs are widely used to avoid gastrorrhagia induced by anti-platelet drugs in patients who suffer from CVD. Accumulation of clinical data uncovered associations between PPI usage and adverse CV event in general population [7,27]. A large study including 396 296 patients confirmed that the risks of first-time ischemic stroke in the general population may be higher in the PPI group in comparison to the non-PPI group [28]. Nitric oxide (NO) has a protective effect on vascular endothelium by reducing the interaction between platelets and endothelium and activating platelets [29]. Studies have shown that PPI inhibits the activity of dimethylarginine dimethylaminohydrolase (DDAH), while DDAH is the catalytic enzyme that predominates the metabolism of asymmetric dimethylarginine (ADMA). The decrease in the DDAH activity reduces the inactivation of ADMA and leads to the accumulation of ADMA in the body. ADMA is an inhibitor of endogenous nitric oxide synthase, which can compete to inhibit the production of NO. The reduction of NO will increase the peripheral vascular resistance, induce oxidative stress, further resulting in inflammation and thrombosis [30] and finally accelerating the development of CVD. A study suggested that after 1 week of PPI treatment, serum ADMA also significantly increased in mice [31]. Yet, the exact mechanism of PPI usage and CVD in PD patients need further research.

This research has several limitations. First of all, as sufficient follow-up data were missing, subgroup analysis on the total duration and dose of PPI usage could not be performed, which lead to potential patient selection bias, but after tracking one of the centers, it was found that the vast majority of patients used PPI continuously for more than 1 month. Secondly, as the patients’ medication prescriptions could not be recorded fully in this study, it was not possible to compare whether there was a difference between the two groups at baseline between the antiplatelet agents such as clopidogrel, tigretol, and indobufen. Therefore, the interaction between PPI and antiplatelet agents could not be further verified in this study. Thirdly, due to excessive lack of blood magnesium data in the database, there was a failure to further confirm the relationship between proton pump inhibitors and hypomagnesemia. Besides, owing to the failure to completely record the cause of morality, it was not possible to further explore the association between PPI and different causes. In addition, the total number of patients in this study was not large enough and therefore there is a need to enroll more multicenter data to verify the conclusions of this study in the future. Finally, since this study was a retrospective analysis, the patient’s medication data could not be fully recorded, and a comparative analysis of H2 receptor blockers had not been performed.

## 5. Conclusions

In this multicenter retrospective research, it was shown that PPI usage was associated with increased risk of all-cause mortality and CV events in PD patients. Although the study could confirm a causal relationship of this conclusion, given the high incidence of abdominal complaints among patients with ESKD, determining PPI utility is challenging and warrants further study.
